# Plasma Glial Fibrillary Acidic Protein and p-Tau217: Potential Biomarkers of Delirium in Older Adults

**DOI:** 10.1093/geroni/igaf122.1055

**Published:** 2025-12-31

**Authors:** Tamara Fong, Julianna Liu, Edward Marcantonio, Richard Jones, Long Ngo, Sarinnapha Vasunilashorn, Sharon Inouye

**Affiliations:** Beth Israel Deaconess Medical Center, Harvard Medical School, Boston, Massachusetts, United States; University of Rochester Medical Center, Rochester, New York, United States; Beth Israel Deaconess Medical Center, Boston, Massachusetts, United States; Brown University, Brown University, Rhode Island, United States; Beth Israel Deaconess Medical Center, Boston, Massachusetts, United States; Beth Israel Deaconess Medical Center, Boston, Massachusetts, United States; Marcus Institute for Aging Research, Hebrew SeniorLife, Harvard Medical School, Boston, Massachusetts, United States

## Abstract

**Objective:**

Delirium is a common complication of hospitalization among older adults, and is associated with cognitive decline, but the underlying mechanisms are poorly understood. Delirium and dementia are closely interrelated; therefore, blood biomarkers for Alzheimer’s disease (AD) and neural injury may yield insight to potential mechanisms in delirium.

**Methods:**

Data were obtained from the Successful Aging after Elective Surgery (SAGES) study (n = 530). Glial fibrillary acidic protein (GFAP), a marker of reactive astrocytosis elevated in neurodegeneration and neural injury, and tau phosphorylated at residue 217 (p-Tau217), a marker of AD pathology, were measured from plasma collected prior to surgery. Post-operative delirium incidence and severity were assessed using the Confusion Assessment Method (CAM) and CAM-S (0-19, 19 worst), respectively.

**Results:**

Higher GFAP (4th vs. 1st quartile) was associated with delirium incidence (relative risk (RR) 1.7, 95% confidence interval (CI): 1.1-2.8) and greater CAM-S severity (adjusted mean difference=0.5, 95% CI: 0.1-1.0). p-Tau217 did not significantly predict delirium incidence, though higher levels were associated with greater severity (adjusted mean CAM-S difference 1st to 4th quartile=0.6, 95% CI: 0.1-1.0).

**Interpretation:**

High pre-operative plasma GFAP was associated with higher delirium incidence and severity, whereas high p-Tau217 was associated only with higher severity. Overall, GFAP appears to be a stronger risk marker than p-Tau217 for delirium. Further work is needed to establish whether astrocytosis contributes to delirium pathophysiology.

